# Biofilm-Forming Ability of *Microbacterium lacticum* and *Staphylococcus capitis* Considering Physicochemical and Topographical Surface Properties

**DOI:** 10.3390/foods10030611

**Published:** 2021-03-13

**Authors:** Elena Zand, Hedwig Pfanner, Konrad J. Domig, Gerhard Sinn, Marija Zunabovic-Pichler, Henry Jaeger

**Affiliations:** 1 Institute of Food Technology, University of Natural Resources and Life Sciences Vienna (BOKU), 1190 Vienna, Austria; elena.zand@boku.ac.at (E.Z.); hedwig.pfanner@students.boku.ac.at (H.P.); henry.jaeger@boku.ac.at (H.J.); 2 Institute of Food Science, University of Natural Resources and Life Sciences Vienna (BOKU), 1190 Vienna, Austria; konrad.domig@boku.ac.at; 3 Institute of Physics and Material Sciences, University of Natural Resources and Life Sciences Vienna (BOKU), 1190 Vienna, Austria; gerhard.sinn@boku.ac.at

**Keywords:** biofilm, *Microbacterium lacticum*, food contact surface, stainless steel, hygienic design, roughness

## Abstract

Biofilm characteristics of *Microbacterium lacticum* D84 (*M. lacticum*) and *Staphylococcus capitis* subsp. *capitis* (*S. capitis*) on polytetrafluoroethylene and AISI-304 stainless steel at early- (24, 48 h) and late-stage (144, 192 h) biofilm formation were investigated. *M. lacticum* biofilm structure was more developed compared to *S. capitis*, representing vastly mature biofilms with a strongly developed amorphous matrix, possibly extracellular polymeric substances (EPSs), at late-stage biofilm formation. *S. capitis* showed faster growth behavior but still resulted in a relatively flat biofilm structure. Strong correlations were found between several roughness parameters and *S. capitis* surface coverage (r ≥ 0.98), and between total surface free energy ($\gamma_{s}$) and *S. capitis* surface coverage (r = 0.89), while *M. lacticum* remained mostly unaffected. The pronounced ubiquitous biofilm characteristics make *M. lacticum* D84 a suitable model for biofilm research. Studying biofilm formation of these bacteria may help one understand bacterial adhesion on interfaces and hence reduce biofilm formation in the food industry.

## 1. Introduction

Biofilms are the most frequent cause of food contamination and thus a leading health and safety concern, as reported by several reviews [1,2,3,4]. They are also linked to chronic infections, as well as foodborne illness outbreaks [5,6]. As biofilms show increased resistance to mechanical, physsical, and chemical treatments, in comparison to their planktonic state [7], it is crucial to select suitable sanitation methods [8]. It is vital to understand that the embedded extracellular matrix of persistent biofilms can protect present potential pathogens and spoilage microorganisms from disinfection treatments [9]. Any persistent microorganism with the ability to form biofilms can lead to human diseases or alteration in sensory characteristics [4].

In biofilm research, also known as “biofilmology” [10], the biofilm-forming ability describes whether the tested microorganism is capable of producing biofilms at the tested growth conditions, including medium composition, temperature, pH and osmolarity [11]. Zou and Liu [12], focusing on bacterial isolates from a milk powder processing facility, classified their tested strains in negative, weak, moderate, and strong biofilm producers.

*Staphylococcus* spp. are Gram-positive, nonspore-forming, and cocci-shaped bacteria, known for strong biofilm formation [13,14]. Staphylococci belong to the most frequently isolated bacteria from food contact surfaces [15,16]. Like *Staphylococcus aureus* (*S. aureus)*, *S. capitis* is a human skin contaminant and a known opportunistic human pathogen [17], being able to grow on the skin or mucous membranes of people. In contact with food, it is a major challenge for the food industry. Different *S. capitis* strains with biofilm-formation potential were previously isolated from poultry and bakery producing environments and showed high resistance against conventional disinfectants, such as chlorhexidine and quaternary ammonium compounds [18]. Besides staphylococci, *Microbacterium* spp. were previously isolated from dairy, egg, ready-to-eat, and meat-producing industries [9,19,20], among which *M. lacticum* is abundant on food contact surfaces in dairy-related facilities [9,19]. *Microbacterium* spp. are Gram-positive, nonsporulating, rod-shaped bacteria. *M. lacticum* adherent to abiotic surfaces is not only found to be persistent against cleaning and disinfection [9] but also against pasteurization [20]. The study by Weber, Liedtke, Plattes and Lipski [9] also reported that isolated *M. lacticum* can lead to contamination and reduced shelf-life of ultra-high-temperature (UHT) processed milk, due to its spoilage- and biofilm-forming potential. Both *S. capitis* and *M. lacticum* were previously studied in their planktonic form, but there was no detailed study on their biofilm-related characteristics.

In principle, bacterial adhesion occurs on any liquid/solid interface, including food contact surfaces, liquid pipelines, or gaskets. Biofilm development includes a first phase, where bacteria reversibly and irreversibly adhere to the interface. Subsequently, microcolonies and a three-dimensional shaped architecture embedded in extracellular polymeric substances (EPSs) forms. After the maturing phase, single motile bacteria detach from the biofilm and may colonize in new trenches, pits and crevices [21,22,23,24,25]. Reasons for biofilm dispersion vary and include limited nutrient availability, fluid shear stress, or enzymatic activities [1].

Since stainless steel (SS) and polytetrafluoroethylene (PTFE) are widely used as food contact materials, they have been often applied in biofilm research [26,27]. SS is preferably used for process equipment due to its high mechanical strength, cleanability, and corrosion resistance. The austenitic SS grades AISI-304 and the low carbon type AISI-304L are considered as corrosion-resistant at ambient temperature, with a pH ranging from 6.5 to 8.0, and low chloride levels. Thermoplastic polymers such as PTFE are used as e.g., conveyor and belt coatings [28]. Due to its sensibility to temperature changes, potentially resulting in brittle cracking, it may become more challenging to clean. Moreover, surface damages due to abrasion or aging can reduce the cleanability of plastic materials and promote biofilm development [29,30].

As bacterial biofilm adhesion and its correlation to interfacial surface characteristics are still controversially discussed, a better understanding is needed to understand the mechanisms of biofilm formation and further to reduce and/or prevent biofilm-associated contaminations [31] in open and closed processes. Therefore, this research aims to mimic the growth behavior of static mono-species *S. capitis* and *M. lacticum* biofilms on food contact surfaces such as SS and PTFE in vitro. Microscopic, physicochemical (hydrophobicity measurements), mechanical (roughness), and culture-based methods were used to analyze interfacial surface characteristics in correlation to biofilm adhesion.

## 2. Materials and Methods

### 2.1. Selected Bacterial Isolates and Culture Conditions

*M. lacticum* D84 (EF204392), isolated from extended shelf-life (ESL) milk, and *S. capitis* subsp. *capitis*, isolated from an air decontamination step prior to packaging at a meat production facility, were selected for biofilm studies, as they are persistent food-related contaminants with less studied biofilm-related characteristics. The isolates were preserved in a 50% (*v*/*v*) glycerol stock at −80 °C. To obtain a stock culture, bacteria were sub-cultured overnight in tryptic soy broth (TSB; Carl Roth, Karlsruhe, Germany) at 37 °C and in 0.8% (*v*/*v*) skimmed milk broth (MB; Carl Roth, Karlsruhe, Germany) at 30 °C, for *S. capitis* and *M. lacticum*, respectively. Subsequently, stock cultures were then streaked onto either tryptic soy agar (TSA) or milk agar (MA) (Carl Roth, Karlsruhe, Germany) and incubated at 37 °C or 30 °C overnight and stored at 4 °C. Before each experiment, one colony was inoculated in 10 mL fresh TSB or MB, and the optical density 600 (OD) was standardized to 0.1 (corresponding to 6.7 ± 0.2 log_10_ colony-forming units (CFUs) per mL^−1^ and 8.5 ± 0.2 log_10_ CFUs mL^−1^ for *S. capitis* and *M. lacticum*, respectively) to obtain a working culture.

### 2.2. Surface Materials and Cleaning Procedure

PTFE coupons (Ø = 22 mm, 1.0 mm thickness; GAMMA Kunststofftechnik GmbH, Vienna, Austria) and austenitic AISI-304 SS coupons (EN 1.4301; Ø = 22 mm, 0.5 mm thickness; Metallvertrieb ds GmbH, Surheim, Germany) were used as abiotic surface materials. SS coupons, consisting of 18% chromium, 10% nickel, and < 0.03% carbon [32], were mechanically polished by 320 and 240 grit (SS 320, SS 240) to achieve different surface roughness. Before each experiment, surfaces were cleaned according to Pérez Ibarreche, et al. [33] with acetone and 1 M of NaOH. Subsequently, coupons were autoclaved.

### 2.3. Biofilm Formation on Model Surface Materials

For biofilm growth on surface materials, 3 mL of the working culture was pipetted into a petri-dish (35 × 10 mm; Greiner Bio-One, Kremsmünster, Austria) and biofilm formation was analyzed up to 192 h, with orbital shaking (60 rpm) at 30 °C for *M. lacticum* or without shaking at 37 °C for *S. capitis*. Non-adherent bacterial cells were removed at 24, 48, 72, and 144 h by gently rinsing twice with 3 mL of phosphate-buffered saline (PBS, pH 7.4, composition: 0.14 M of NaCl, 2.7 mM of KCl, 10 mM of phosphate; Carl Roth, Karlsruhe, Germany). The biofilms were incubated accordingly.

### 2.4. Culture-Based Analysis of Biofilms

The biofilm growth was enumerated at 24, 48 144, and 192 h by the drop plating method. For cell detachment efficiency, scraping and sonication were compared. For scraping (mechanical action), coupons were washed twice in PBS, resuspended in 1 mL of PBS and scraped off four times vertically and horizontally by using a sterile pipette tip (Figure S1). The solution was centrifuged at 4000× *g* for 10 min. Subsequently, after tenfold serial dilution, samples were drop plated (6 × 5 µL; 10^−1^–10^−6^) onto TSA (37 °C, 24 h) or MA (30 °C, 72 h) and expressed as log_10_ CFU cm^−2^. For the sake of simplicity, log_10_ will be referred to as *log* hereafter. For the sonication (ultrasound; chemical and physical action), the coupons were washed, resuspended in 1 mL of PBS and treated three times for 1 min at 35 kHz (Ultrasonic bath, Sonorex RK100H; Bandelin electronic, Berlin, Germany), as described by Schulte [34] and drop plated. The treatment time for cell removal was assessed in pre-trials to exclude cell injury by sonic waves (data not shown). For both methods, a minimum of three biological and technical triplicates was performed.

### 2.5. Surface Coverage and Cell Viability with Epifluorescence Microscopy (EFM)

The biofilms were washed and stained with 3.34 µM of SYTO^TM^ 9 (Thermo Fisher Scientific, Waltham, MA, USA) and 20.00 µM of propidium iodide (PI; Thermo Fisher Scientific, Waltham, MA, USA) in TSB and incubated for 20 min at 37 °C in the dark, as described by Rodríguez-Melcón, et al. [35]. The surface coverage and viability of biofilm formation on surface materials was quantified at 24, 48, 144, and 192 h using a stereomicroscope (Olympus SZX16; Olympus, Vienna, Austria). Measurements were conducted using two filter cubes, with an excitation filter of BP470/40 and BP545/30, for SYTO^TM^ 9 and PI, respectively, with a magnification of 7× and 100×. The quantitative area of cells with an intact cell membrane (viable; green fluorescence) and membrane-comprised cells (dead; red fluorescence) was analyzed using ImageJ/NIH image software (Version 1.48v) [36]. Negative controls of surface materials and nutrient media without staining procedure were included and subtracted from the final results. Measurements were performed in biological and technical triplicates.

### 2.6. Descriptive and Structural Evaluation of the Biofilm Matrix by Scanning Electron Microscopy (SEM)

For the structural analysis of exemplary early and late biofilm growth with SEM (Quanta^TM^ 250 FEG; FEI, Hillsboro, OR, USA), biofilm formation at 48 and 192 h was considered. After biofilm growth on small SS and PTFE coupons (10 × 10 mm, l × w), fixation and dehydration steps of biofilm specimens were performed, as described by Murtey and Ramasamy [37] but without postfixation. Fixation was carried out overnight in a 2.5% (*v*/*v*) glutaraldehyde solution at 4 °C. After ascending dehydration steps with an ethanol and 25% hexamethyldisilazane solution, the samples were dried in a desiccator at 20 °C for 16 h. Before SEM analysis, the specimens were mounted on an SEM sample stub and gold-coated (HHV Scancoat Six; HHV Ltd., Crawley, United Kingdom). Specimens were scanned with an emission current of 216–230 µA and a high vacuum (chamber pressure: 0.1 mbar) at an accelerating voltage of 20 kV and 10.0 mm WD or 10 kV and 5.5 mm WD, for SS or PTFE specimen, respectively. Images of biological duplicates were taken at magnifications of 1000×, 5000×, 10,000×, 30,000×, and 55,000×.

### 2.7. Surface Topography Measurement

The areal surface roughness and line roughness, according to ISO 25178-2:2012 [38] and BS EN ISO 4288:1998+A1:2009 [39] was defined using a three-dimensional CLSM (VK-X1000; Keyence International, Mechelen, Belgium). For the areal surface texture, which is an extension of the line profile roughness, the mean arithmetic height of the surface roughness (S_a_), the maximum height (S_z_), the root mean square height (S_q_), the maximum pit height (S_v_) and the maximum peak height (S_p_) were assessed. For line roughness, R_a_, the mean roughness depth (R_z_), R_q_, the maximum depth of valleys (R_v_), the maximum height of peaks (R_p_) and RS_m_, a line roughness spacing parameter, were included. For line roughness parameters, 41 measurements were performed, while for S_a_- and S_z_-values, one representative surface material was analyzed. It has to be noted that mechanical interactions during the grinding of steel surfaces, like for AISI 304 SS, cause surface roughness anisotropy [40]. In this study, only roughness parameters perpendicular to the main surface structure were considered.

### 2.8. Physicochemical Surface Properties

Contact angle measurements were performed with a drop shape analyzer (Digidrop; Fa. GBX, Romans-sur-Isère, France) at room temperature by the sessile drop method. The analysis was carried out to investigate surface hydrophobicity, also known as wettability. For each measurement, with the help of a microsyringe, ~5 µL of liquid was carefully dropped on the surface. For pure contact angle measurements, MB and TSB were applied. For steel surfaces, all contact angles were measured perpendicular to the main surface structure.

For surface free energy calculation, reference liquids, including diiodomethane, formamide (both >99.5% purity, VWR International, Radnor, PA, USA), and dH_2_O, were used. Droplets were placed on the specimens, and time series of ten seconds were recorded. Every 40 ms, the right and left contact angles were evaluated and averaged. A minimum of 4 drops was placed on two independent surfaces. Surface free energy parameters were calculated based on the acid-base approach according to Van Oss, et al. [41]. The work of adhesion can be described as follows and gives the formal connection of the contact angle with the surface free energy components:

(1) $$\gamma_{L}(1+\cos\;\theta )=2\left[\left(\sqrt{\gamma_{S}^{LW}\gamma_{L}^{LW}}\right)+\left(\sqrt{\gamma_{S}^{+}\gamma_{L}^{-}}\right)+\left(\sqrt{\gamma_{s}^{-}\gamma_{L}^{+}}\right)\right]$$

the total surface free energy $(\gamma_{s})\;$consists of the Lifshitz-van der Waals component $\left(y_{s}^{LW}\right)$ and the polar Lewis acid–base component $\left(y_{i}^{AB}\right)$. $y_{i}^{AB}$consists further of the electron-acceptor $(\gamma_{s}^{+})\;$and -donor parameter $(\gamma_{s}^{-})\;$ [42,43]; $\gamma_{L}$ is the surface tension of the liquid:

(2) $$y_{S}=y_{s}^{LW}+\;y_{i}^{AB}$$

(3) $$y_{i}^{AB}=2\;\sqrt{\gamma_{s}^{+}\gamma_{s}^{-}}$$

### 2.9. Statistical Analysis

Results are presented as the mean value ± standard deviation. For statistical significance, a multifactor ANOVA with two independent factors and Tukey’s t-test were carried out in Statgraphics Centurion XVIII (Statpoint Technologies, Inc., Warrenton, FL, USA). A statistical significance was considered at *p* < 0.05. For goodness-of-fit statistics, the regression coefficient r^2^ was measured. The linear correlation coefficient (r) was used to obtain the relationship between biofilm growth or surface coverage to surface characteristics. Data were visualized with Sigma Plot 13 (Systat Software, Inc., San Jose, CA, USA).

## 3. Results and Discussion

### 3.1. Comparison of the Bacterial Cell Detachment Approaches

According to the literature, different approaches are used for cell detachment, including scraping with a sterile pipette tip [44,45] or stick [46], sonication [34,47,48,49], wet-swabbing [50] or the violent water-flapping method, introduced by Wang, et al. [51].

**Figure 1:**
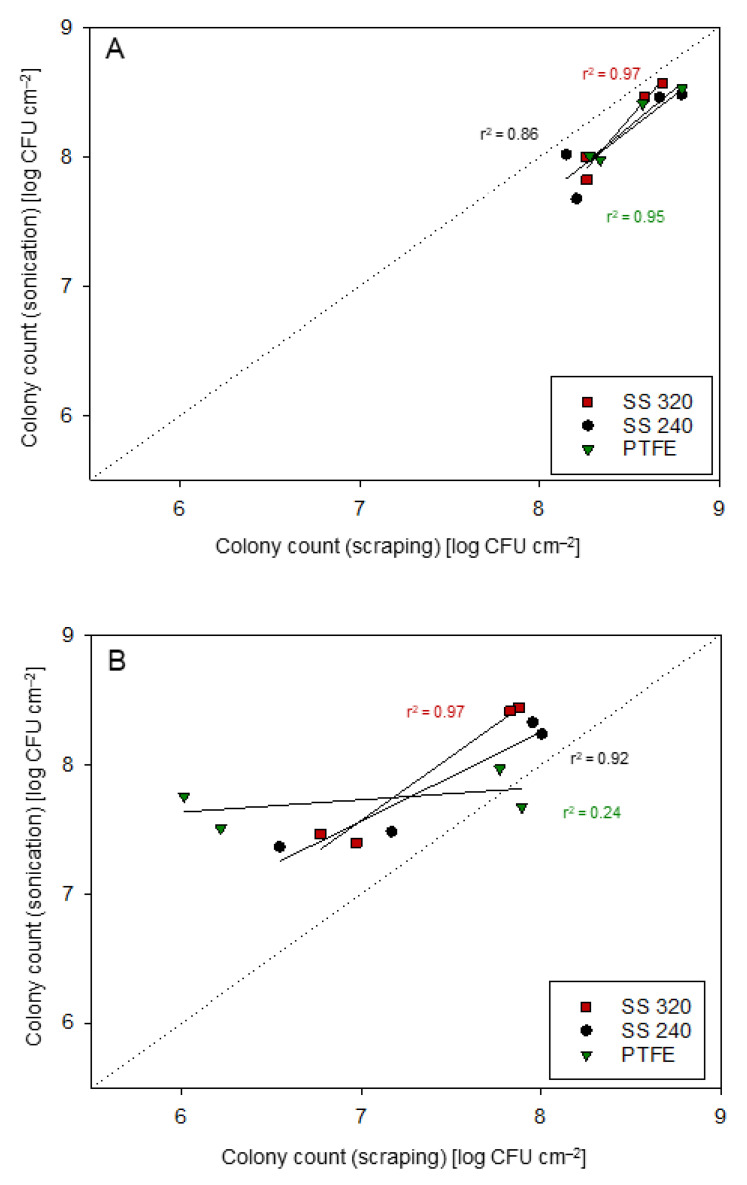
Goodness-of-fit statistics of the cell detachment methods sonication (y-axis) and scraping (x-axis) for *M. lacticum* (**A**) and *S. capitis* (**B**). Data points represent mean values. CFU: colony-forming unit.

As it is crucial to use reproducible methods, the present study compared scraping and sonication for *S. capitis* and *M. lacticum* biofilm formation at the early- (24–48 h) and late-stage (144–192 h) (Table S1). Goodness-of-fit statistics (r^2^ ≥ 0.92; Figure 1) indicated high agreement with *M. lacticum* or *S. capitis* colony counts obtained by scraping and sonication, except for *M. lacticum* growth on SS 240 and *S. capitis* growth on PTFE. For *M. lacticum* on SS 240, the growth difference (≤0.5 log CFU/mL) between both methods was within the normal range of experimental error and thus not further considered. In contrast, *S. capitis* growth on PTFE at the early-stage was significantly (*p* < 0.05) different between the two methods, indicating higher counts after sonication. It is assumed that either scraping underestimated *S. capitis* counts on PTFE or that the biofilm in these experiments developed poorly, containing a lower quantity of cells. Bjerkan, et al. [52] concluded that sonication efficiently removed biofilm cells from surfaces, while different scraping approaches used in their study removed fewer cells and resulted in inconsistent findings. In contrast, Kragh, et al. [53] reported that scraping in schematic order with a sterile pipette tip removed significantly more cells from a well (90% of attached cells) than sonication. In the present study, detachment by scraping showed very consistent findings (experimental error ≤0.4 log CFU/mL), even though it is based on mechanical action. Scraping was also more in line with data obtained by SEM and quantitative EFM measurements than detachment by sonication. Hence, the results presented in the following Section 3.2. are based on the scraping method.

### 3.2. Culture-Based Enumeration of Biofilm Development Using the Scraping Technique

**Figure 2:**
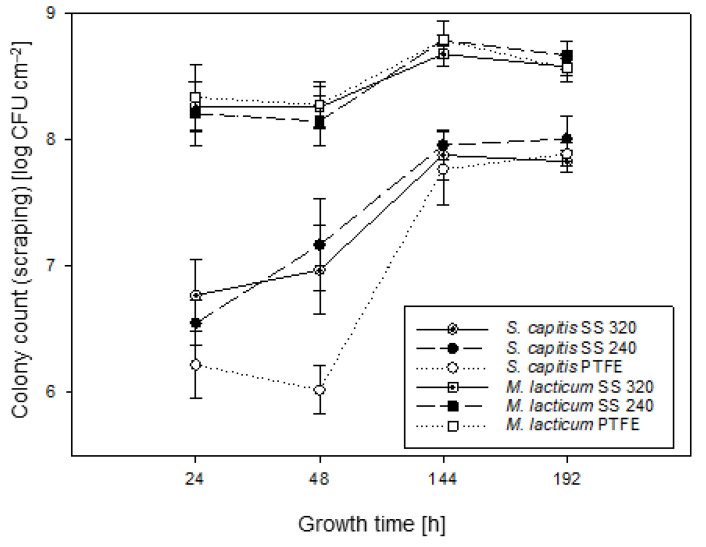
Growth curves of *M. lacticum* (□, ■, ◘) and *S. capitis* (○, ●, ⦿) biofilms at 24, 48, 144, and 192 h on stainless steel (SS) 240, SS 320, and polytetrafluoroethylene (PTFE) surfaces, obtained by the scraping technique. For culture-based analysis, mean values ± standard deviation are based on three independent experiments.

**Figure 3:**
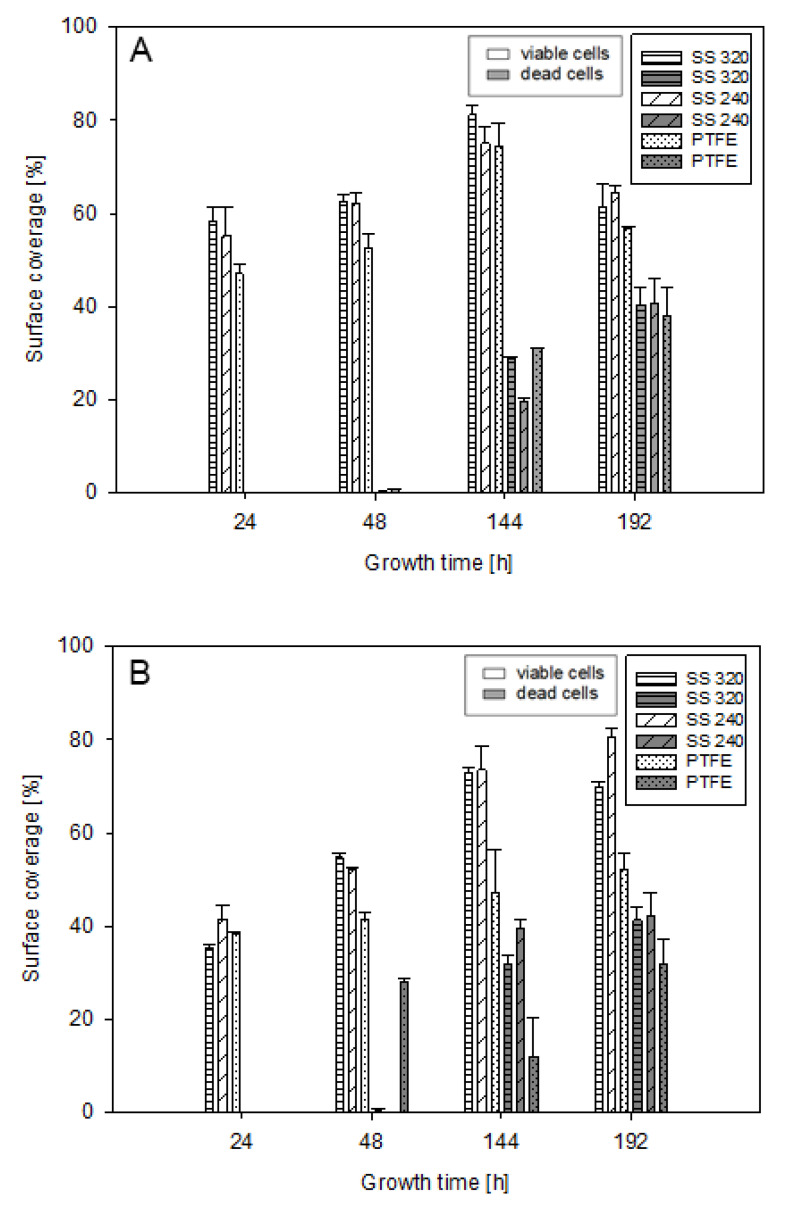
*M. lacticum* (**A**) and *S. capitis* (**B**) surface coverage (%) at 24, 48, 144, and 192 h through cell staining with SYTO^TM^ 9 and propidium iodide (PI) on PTFE and SS coupons. The proportion of viable (white bars; SYTO^TM^ 9) and dead cells (grey bars; PI) is indicated. For image analysis, an area of 260 mm² was investigated.

Figure 2 illustrates the biofilm growth behavior of *M. lacticum* and *S. capitis*, removed from the substrate by scraping. Overall, the extent of *S. capitis* biofilm growth (max. 7.2 log CFU cm^−2^) at the early-stage (24–48 h) was similar to previous findings for *S. carnosus* at 48 h (max. 6.6 log CFU cm^−2^) [54] and *S. aureus* biofilms at 240 h (max. 6.3 log CFU cm^−2^) [55]. For *M. lacticum* biofilm growth, a slight peak at 144 h (max. 8.8 log CFU cm^−2^) and a subsequent slight decrease in log CFU cm^−2^ was observed. Together with EFM data (Section 3.3, Figure 3), this leads to the assumption that at 192 h, single bacteria already detach from the biofilm, resulting in the dispersal phase. In contrast, for *S. capitis* biofilm growth, the highest log CFU cm^−2^ within the investigated period of time was observed at 192 h (max. 8.0 log CFU cm^−2^). These findings indicate fully mature biofilms at 144 h and 192 h (late-stage), for *M. lacticum* and *S. capitis*, respectively. In the study of Iñiguez-Moreno, et al. [56], several mono-species biofilms resulted in full maturity on SS and polypropylene surfaces at 240 h, resulting in a growth of ≥7.7 log CFU cm^−2^. These findings are in agreement with late-stage biofilm formation in the present study. The present findings showed that *M. lacticum* biofilm growth was significantly more developed (*p* < 0.05) than *S. capitis*, even though for *S. capitis,* a trend towards faster growth was indicated.

If one considers the growth curves solely, the tested surface materials did not significantly (*p* < 0.05) affect the biofilm growth. Only bacterial growth on PTFE after 48 h was significantly lower (*p* < 0.05) compared to SS surfaces. Likewise, bacterial attraction to specific interfaces was controversially discussed in the literature. Planchon, Gaillardmartinie, Leroy, Bellonfontaine, Fadda and Talon [54] found a preferred growth of *S. carnosus* biofilms on SS (AISI-304) compared to PTFE surfaces. Other researchers suggested that *Staphylococcus* spp. attach and stick very well to plastic surfaces [57,58,59]. The latter was related to autolysins [17]. Several studies linked specific surface characteristics, including roughness or hydrophobicity, to cell adhesion, which is discussed in Section 3.5 and Section 3.6.

At the time of research, there was no other study focusing on *M. lacticum* biofilm growth characteristics. Comparative discussion for the observed strain was, therefore, quite challenging. In the literature, protease activity was considered as a promotor for the strong biofilm formation of *M. lacticum* [9]. Vithanage, et al. [60] found protease, lipase, or phospholipase C activity in over 80% of *Microbacterium* raw milk isolates. Those enzymes are suggested to have different key roles within the biofilm formation process. The ubiquitous and strong biofilm growth of *M. lacticum*, independent of substrate properties or temperature changes [20], as well as its increased resistance to disinfectants [9], render *M. lacticum* as a highly persistent challenge for the food industry.

### 3.3. Surface Coverage and Biofilm Viability

**Figure 4:**
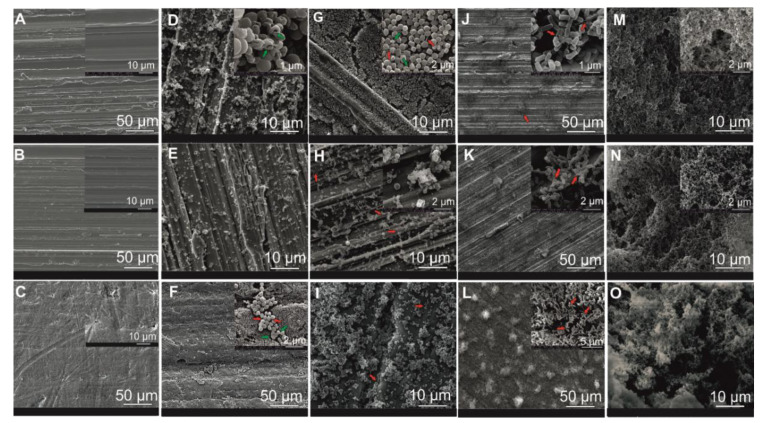
Representative scanning electron microscopy (SEM) images of the reference surface materials SS 240 (**A**), SS 320 (**B**), PTFE (**C**), and the biofilm structure. *S. capitis* biofilm at 48 h on SS 240 (**D**), SS 320 (**E**), and PTFE (**F**) and at 192 h on SS 240 (**G**), SS 320 (**H**) and PTFE (**I**). *M. lacticum* biofilm at 48 h on SS 240 (**J**), SS 320 (**K**), PTFE (**L**), as well as at 192 h on SS 240 (**M**), SS 320 (**N**), and PTFE (**O**). The amorphous matrix (possibly an extracellular polymeric substance (EPS); red arrows) and membrane-compromised cells (green arrows) are visualized in color.

Surface coverage and viability of *M. lacticum* and *S. capitis* cells were assessed at early- (24–48 h) and late-stage (144–192 h) biofilm formation (Figure 3). The proportion of viable intact cells, covering the observed area of 260.55 mm^2^, ranged from 35–81% for *S. capitis* biofilms and from 47–81% for *M. lacticum* biofilms. Hence, indicating the same maximum proportion of viable cells at late-stage biofilm formation. For *M. lacticum*, dead cells resulted in ≤1% and 29–41%, and for *S. capitis* in 0–28% and 12–42%, at early- and late-stage biofilm formation, respectively. Late-stage biofilm formation showed an overall significant (*p* < 0.05) increase in impaired cells in comparison to biofilm formation at the early-stage. Solely for *S. capitis* on PTFE, there was no significant difference (*p* > 0.05), as a relatively high amount of dead cells (28%) was already observed at 48h. Together with the descriptive SEM data (showing compromised cell membranes; Section 3.4, Figure 4F) and its lower lag phase, as indicated in the growth curve (Section 3.2, Figure 2), inferior growth conditions for *S. capitis* on PTFE surfaces are assumed. Therefore, *S. capitis* cells on PTFE require a more prolonged adaptation phase for biofilm formation. verall, the biofilm viability and surface coverage underlined the biofilm maturity of *M. lacticum* and *S. capitis* at late-stage biofilm formation, as previously suggested from culture-based analysis. The increased amount of dead cells at late-stage biofilm formation is in agreement with previous findings for *Listeria monocytogenes (L. monocytogenes)* [61] and *Staphylococcus* spp. biofilms [17,62]. It is known that individual bacterial cells can deliberately exceed their exponential growth phase in order to achieve a growth decline or die. This stress response mainly occurs as nutrients diminish and it enables cells to contribute to the preservation of the biofilm structure as well as to protect the system from energy loss [17,61,62]. Moreover, damaged or dead cells usually contribute to a greater extent to outer layers of the biofilm, which is known as a protection mechanism [63].EFM as an analytical method might over- or underestimate the proportion of live and dead cells, as the method only supports two-dimensional images, while biofilms have complex three-dimensional structures. With flow cytometry as an alternative approach for the viability analysis of biofilms, it is possible to elucidate this error as cell aggregates are dissolved, and single cells inside the biofilm are measurable [64]. If studying biofilms on transparent surfaces, a detailed analysis of the three-dimensional biofilm structure, including its viability, can be performed with confocal laser scanning microscopy [65]. Considering the direct staining of the cells, biases such as the background fluorescence of PI in the unbound form or bleaching of SYTO^TM^ 9, especially during SYTO^TM^ 9-PI staining, can occur [66]. Another limitation is that the nutrient media [67], surface materials [68], or cells themselves [69] can emit autofluorescence and thus, false-positive results can occur. SS is known as non-autofluorescence substrate [68] in the nanometer range observed within this study. Besides the surface materials, the nutrient broths, such as TSB or MB, could have slightly affected the results, even though negative controls were included and subtracted from samples. Such unwanted signals, also known as background or noise that do not represent fluorescence, are a fundamental challenge for fluorescence-based assays [69] and could explain why the percentage of total surface coverage (=viable and dead cells) was sometimes higher than 100%. EFM is a supportive analysis for viability and morphology but may not be suitable as a standalone method for biofilm research.

### 3.4. Qualitative Structural and Morphological Biofilm Analysis

In Figure 4, SS 240, SS 320, and PTFE surface topography as well as microstructural *M. lacticum* and *S. capitis* biofilm formation at both the early- and late-stage (48 h and 192 h), are visualized.

At 48 h, adhered *S. capitis* formed evenly distributed microcolonies, resulting in a flat and patchy structure (Figure 4D–F). Their appearance as grape-like clusters was most evident on PTFE coupons, while growth on SS 240 suggested the first three-dimensional structures. At 192 h, cells indicated different appearances on the three surface materials (Figure 4G–I). On SS 240, cells almost completely covered the surface, showing a very dense structure, while on PTFE, the three-dimensional structure appeared more pronounced, but the surface was not yet covered. Notwithstanding, only weak amorphous matrices, possibly EPSs, have formed. Besides, damaged cells, characterized by a rough and shrunken appearance [44], were visible in some of the *S. capitis* close-up views (Figure 4D,F–H; green arrows) and are in agreement with quantified dead cells obtained with EFM (Section 3.3, Figure 3). Cell-to-cell interactions are envisioned in the close-up view of SS 240.

The *M. lacticum* growth resulted in three-dimensional structures at 48 h (Figure 4J–L), most evident on PTFE surface. In the close-up views of *M. lacticum* (Figure 4J–L, red arrows), amorphous matrix, fibers, and amorphous substances, most likely EPSs, were evident. EPSs were generally observed as thin fibers or filaments, as the extracellular matrix, consisting of 95% water, collapsed due to dehydration during SEM sample preparation [70]. At 192 h, a very thick and stable *M. lacticum* biofilm matrix with a relatively homogenous solid amorphous matrix covered the surface materials (Figure 4M–O).

Overall, *M. lacticum* produced a mature and rigid biofilm, while *S. capitis* biofilms were indicated as flat and pliable structures. The amorphous matrix, possibly an EPS, was vastly more developed for *M. lacticum* than for *S. capitis*. Growth curves also showed a slight increase in *M. lacticum* compared to *S capitis* growth, but colony counts in the late-stage biofilm formation resulted in a marginal difference only (<1 log CFU/cm^−2^; Section 3.2, Figure 2). This highlights that the structural analysis of biofilms is essential in addition to culture-based analysis, as the latter does not consider the biofilm morphology and structure and might lead to false predictions about the biofilm-forming ability if used as a standalone method. Besides morphological observation, EPS production can be quantified with techniques such as the phenol–sulfuric acid method [71].

The structural appearance of *S. capitis* is was comparable to other findings for *S. aureus* biofilms at 48 h and 72 h on polystyrene plates [44]. The present findings of *M. lacticum* were compared to other genera, as studies on *M. lacticum* biofilms are very limited. Observations for *M. lacticum* at 48 h were in agreement to findings for *Lactobacillus sakei* biofilms on SS and PTFE at 6 d (144 h) [33], while its appearance at 192 h was similar to images for *L. monocytogenes* on rubber surfaces [72]. Interestingly, *M. lacticum* biofilm growth was also similar to a multi-species biofilm consisting of typical food-related biofilm formers [56]. In a study by Techer, Jan, Gonnet, Grosset, Gautier and Baron [20], *Microbacterium* spp. and *Staphylococcus* spp., isolated from food contact surfaces, showed similar adhesion behavior to SS (AISI-304). Hence, the highly developed biofilm-forming characteristics of *M. lacticum* on the tested food contact materials are probably not related to its rapidity in adhesion and growth. It is suggested that *M. lacticum* D84 is a suitable biofilm strain for further application studies.

For both biofilm formers, different appearances on SS and PTFE surfaces were observed, which can be explained as follows. An adaptation of the extracellular matrix to the substratum topography can cause discrepancies. According to Pérez Ibarreche, Castellano and Vignolo [33], the microcolonies on SS cover all scratches, which results in an overall smoother appearance of their microstructure, while in contrast, on overall flat PTFE, a more amorphous extracellular mass appears. Artifacts can also occur due to material reflection. Besides, the high accelerating voltages used during SEM measurements can have also negatively affected the structural appearance of the biofilm structure [73]. However, similar accelerating voltages, as used in the present study, were previously reported for biofilm research [44,71].

### 3.5. Effect of Surface Roughness on Biofilm Formation

As a recent review concluded, no clear correlation has yet been found between biofilm growth and surface topography [31]. Still, according to EHEDG Document No. 8 [74], the use of surfaces with R_a_-values < 0.8 µm is recommended for food process equipment to realize improved cleanability and prevent bacterial contaminations. However, the effect of surface roughness on bacterial adhesion has been controversially discussed. It should also be noted that surfaces with similar roughness parameters can still have significantly different topographies [75]. Hence, roughness may not be used as a surface indicator for hygienic design properties and cleanability by itself.

In the present study, both line roughness (R_a_, R_z_, R_q_, R_v_, R_p_, RS_m_) [76] as well as areal surface texture parameters (S_a_, S_z_, S_q_, S_v_, S_p_), were considered (Table 1). The R_a_-value defines the height deviation of an area but does not differentiate between valleys, peaks, or spatial structures [29] and is not sensitive to minor alterations. The root mean square roughness (R_q_) is more sensitive to deviations than the R_a_-value and thus a vital roughness parameter [76]. SS 240 was the overall roughest material with an R_a_-value of 1.0 µm and S_a_-value of 1.1 µm, while PTFE was the smoothest substrate, if referring solely to R_a_- and S_a_-values (R_a_: 0.2 µm, S_a_: 0.5 µm). The differences between the line and areal surface parameters are reasonable, as measurements differ [77]. The R_q_ and S_q_ values in the present study were in line with the R_a_ and S_a_ values and thus, were not further considered. Moreover, the RS_m_-value (also known as a spacing parameter), which indicates the average distance between profile peaks on the centerline [76], was comparable on all three tested surface materials, ranging from 9.7–10.7 µm. For PTFE coupons, the high S_z_-value (15.1 µm) combined with the relatively low S_a_-value (0.5 µm) underlined surface irregularities such as pits and scratches. Besides, a high maximum depth of valleys (R_v_) and a high maximum height of peaks (R_p_) could positively affect the bacterial attachment to a surface since protection against external forces is increased. In the present study, the highest values for R_v_ (1.7 µm), R_p_ (2.1 µm), S_v_ (9.7 µm), and S_p_ (5.1 µm) were observed for SS 240.

For *S. capitis*, the different roughness parameters, except for S_z_, positively correlated with the average amount of viable intact cells covering the surface (r ≥ 0.98), further referred to as surface coverage, indicating increased surface coverage on surfaces with higher roughness values (Figure 3, Section 3.3). The preferred growth on SS 240 was also assumed from graphical SEM data (Figure 4, Section 3.4). Previous studies were in agreement and reported that surface topographical dimensions comparable with microbial cell size (~1–2 µm) were found to positively influence cell–surface interaction and the attachment of Gram-positive bacteria to surface materials [31,78,79,80]. However, the present findings also show that *S. capitis* biofilm growth on agar plates was not significantly affected by the surface topography (Figure 2, Section 3.2). For *M. lacticum*, a negative correlation of S_z_ to its surface coverage was observed (r = −0.95). Despite this, only weak or no correlation of surface properties on *M. lacticum* surface coverage or biofilm growth was found, indicating ubiquitous growth behavior. Based on the present study, it is not apparent whether there is a decisive roughness parameter or threshold reducing or even preventing biofilm growth.

The findings of previous research were also controversial. Several studies observed no significant effect of roughness on bacterial attachment and biofilm growth of, e.g., *S. aureus*, *L. monocytogenes* and *Salmonella* spp. [26,81,82], while others found a positive correlation [83,84]. Interestingly, Awad, et al. [85] observed an improved attachment of *Pseudomonas aeruginosa* at higher (R_a_: 0.2 µm) compared to lower surface roughness (R_a_: 0.1 µm) at 4 h, but did not find any correlation of roughness and adherence at a later biofilm formation stage. Thus, the lack of time points below 24 h in the present work may explain why there was no overall correlation between surface roughness and culture-based analysis.

### 3.6. Effect of Physicochemical and Interfacial Properties on Biofilm Formation

In this work, physicochemical surface characteristics, including contact angles and surface free energy parameters, were studied (Table 2). The surface free energy is caused by solid–liquid interactions at an interface, such as van der Waals forces, hydrogen bonding, and Lewis acid–base interactions [86]. It is consequently suitable for investigating the attachment of liquid bacterial biofilms to a solid surface. Surface hydrophobicity or rather wettability is also vital for cleanability [32].

In order to investigate surface wettability, water contact angles were used [87]. According to Vogler [88], a contact angle of >65° is considered as hydrophobic, while everything below is categorized as hydrophilic. Based on this approach, all surfaces in the present study were hydrophobic (*θ*_W_ > 65°); among them, PTFE was the most hydrophobic one (*θ*_W_ = 94.5°). The surface free energy parameters, $\gamma_{s}$ and $y_{s}^{LW},\;$were highly consistent for SS 320 (41.3 mJ m^−2^; 41.0 mJ m^−2^), and SS 240 (40.7 mJ m^−2^; 38.9 mJ m^−2^). PTFE showed lower $\gamma_{s}$ and $y_{s}^{LW}$ (23.5 mJ m^−2^; 21.2 mJ m^−2^), compared to SS 320 and SS 240. Low $y_{i}^{AB}$ values of all three surfaces indicate apolar surface areas, which is consistent with the *θ*_W_-values > 65 °. For SS and PTFE, low electron activity was found. Solely SS 320 showed quite high electron-donating activity, which is comparable to other findings for SS [89]. Overall, results for SS [26,43,87,89,90] and PTFE [43,91,92] agree with the previous literature. Chia, Goulter, McMeekin, Dykes and Fegan [26], Ammar, Swailes, Bridgens and Chen [83] found surface energy values ($y_{s}^{LW},\;\gamma_{s})\;$for PTFE different from the one in the present study.

A positive relationship between $y_{s}^{LW}$ or $\gamma_{s}$ to *S. capitis* surface coverage (r = 0.92, r = 0.89), respectively, was found. Hence, the decrease in surface coverage on PTFE is not only related to roughness parameters but also to its low surface free energy (Figure 3, Section 3.3). Similar results were previously reported by Chia, Goulter, McMeekin, Dykes and Fegan [26], Pereni, Zhao, Liu and Abel [92] for different Salmonella strains and *P. aeruginosa*, respectively. The differences in *S. capitis* biofilm growth on the three surfaces, however, are neglectable (Figure 2, Section 3.2). For *M. lacticum*, no strong correlation between $y_{s}^{LW}$ or $\gamma_{s}$ to biofilm growth or surface coverage were observed, indicating its ubiquitous growth, as mentioned previously.

Besides surface wettability, cell hydrophobicity was discussed to play a vital role in the adhesion process [31]. According to Hood and Zottola [93], most bacterial cells are hydrophobic and preferably adhere to hydrophobic interfaces, while the hydrophilic cells preferably adhere to hydrophilic interfaces [94]. Several studies, however, did not find a relationship between surface hydrophobicity and bacterial attachment or biofilm adhesion [8,26,56,81,95]. Cell hydrophobicity was not examined in the present study but could have affected the relationship between biofilm growth or surface coverage and surface properties. Other potential differences in cell characteristics [31] might also explain the overall more significant effect of surface properties on *S. capitis* surface coverage, compared to *M. lacticum*.

In the present study, contact angles of the applied nutrient media (*θ*_TSB_, *θ*_MB_) were also analyzed to investigate the effect of the selected media on the relationship between biofilms and surface material. Interestingly, *θ*_MB_ (94.5–60.1°) was generally higher compared to *θ*_TSB_ (76.3–54.6°), indicating increased hydrophobic reactivity using MB media. Even though clean surfaces were used for this study, MB media could imitate residual food matrices containing milk powder. During food processing, food contact surfaces are rapidly covered with organic layers of i.e., milk or meat matrix [27]. Whenever a material surface is exposed to a fluid medium containing nutrients, its surface characteristics become frequently modified by the ambient liquid, as organic molecules are adsorbed [89,90]. The increased hydrophobic interaction related to MB media might have positively affected the biofilm formation of *M. lacticum*, compared to *S. capitis* in TSB, and should be considered for the selection of food contact materials in combination with the processed food matrix. This finding is in agreement with Hamadi, Asserne, Elabed, Bensouda, Mabrouki and Latrache [89], who found a positive relation between hydrophobicity and the number of adherend cells. Still, further research is needed to prove our hypothesis for contact angles with nutrient media as an additional reliable parameter in studying biofilm formation of liquid–solid interfaces.

## 4. Conclusions

The present study underlines that a combination of image-based and culture-based methods is essential to comprehensively research biofilm-forming ability, while one method alone may give false predictions. Additionally, surface characteristics are key to better understand and efficiently control biofilm formation on food contact surfaces. *S. capitis* biofilm was affected by several roughness parameters as well as $\gamma_{s}$ and $y_{s}^{LW}$, while *M. lacticum* growth remained unaffected. Independent from the surface characteristics, biofilm formation of *M. lacticum* was more developed than that of *S. capitis*, even though *S. capitis* showed a faster growth behavior. It is hypothesized that the *M. lacticum* growth could have been positively affected by the increased hydrophobicity of the nutrient media used. Therefore, besides the bacteria strain and surface material, the impact of the growth media or residual food matrix should be considered. Striking recommendations such as the Ra-value of <0.8 µm are helpful for cleanability in industrial applications, but it is not clear whether there is a threshold value or specific roughness parameter for preventing biofilm growth. To conclude, this study not only contributes to the understanding of biofilm formation on food contact surfaces but may also help to update hygiene-related guidelines for the food industry.

Further research needs to fully understand biofilm-forming ability on surfaces and include a detailed quantification and characterization of the produced EPS and three-dimensional analysis of biofilm architecture by CLSM.

## Figures and Tables

**Table 1:** Surface topography, including line roughness (R_a_, R_z_, R_q_, R_v_, R_p_, RS_m_) and surface roughness (S_a_, S_z_, S_q_, S_v_, S_p_) parameters of SS 320, SS 240 and PTFE ^a^.

Material	Line Roughness [µm] ^b^						Surface Roughness [µm] ^c^				
	R_a_	R_z_	R_q_	R_v_	R_p_	RS_m_	S_a_	S_z_	S_q_	S_v_	S_p_
SS 320	0.5 ± 0.0	2.5 ± 0.1	0.7 ± 0.0	1.3 ± 0.1	1.5 ± 0.1	10.2 ± 0.5	0.7	7.5	0.6	2.7	2.9
SS 240	1.0 ± 0.0	4.2 ± 0.1	1.0 ± 0.1	1.7 ± 0.1	2.1 ± 0.1	10.7 ± 1.3	1.1	17.6	1.0	9.7	5.1
PTFE	0.2 ± 0.0	0.9 ± 0.2	0.3 ± 0.1	0.5 ± 0.1	0.4 ± 0.1	9.7 ± 2.2	0.5	15.1	0.5	6.9	3.2

^a^ SS 320 and SS 240, Stainless steel with a grit of 320 and 240; PTFE, polytetrafluoroethylene; R_a,_ arithmetic mean roughness value; R_z,_ mean roughness depth; R_q_, root mean square deviation; R_v_, maximum depth of valleys; R_p_, the maximum height of peaks; RS_m_, mean spacing at the mean line; S_a,_ mean arithmetic height of surface roughness; S_z,_ the maximum height of surface roughness; S_q_, root mean square height; S_v_, maximum pit height; S_p_, maximum peak height; ^b^ Means ± standard deviation of 41 line roughness measurements; ^c^ for surface roughness single measurements were performed.

**Table 2:** Physicochemical surface properties of SS 320, SS 240 and PTFE ^a^.

Material	Contact Angle (°) ^b^					Surface Free Energy (mJ m^−2^) ^c^				
	*θ*_TSB_	*θ*_MB_	*θ*_W_	*θ*_D_	*θ*_F_	$\gamma_{s}$	$y_{s}^{LW}$	$\gamma_{s}^{AB}$	$\gamma_{s}^{+}$	$\gamma_{s}^{-}$
SS 320	54.6 ± 1.3	61.4 ± 3.1	65.9 ± 11.7	36.9 ± 5.5	52.7 ± 6.3	41.3 ± 0.4	41.0 ± 0.2	0.3 ± 0.2	0.0 ± 0.0	17.1 ± 0.3
SS 240	58.9 ± 3.2	60.1 ± 4.7	85.6 ± 7.9	41.4 ± 1.1	55.7 ± 8.2	40.7 ± 0.2	39.0 ± 0.1	1.8 ± 0.1	0.5 ± 0.0	1.7 ± 0.1
PTFE	76.3 ± 7.9	94.5 ± 4.5	94.5 ± 8.0	72.8 ± 10.8	66.5 ± 5.8	23.5 ± 0.3	21.2 ± 0.1	2.4 ± 0.1	2.9 ± 0.1	0.5 ± 0.0

^a^ SS 320 and SS 240, Stainless steel with a grit of 320 and 240; PTFE, polytetrafluoroethylene; *θ*_TSB_, contact angle measured with tryptic soy broth; *θ*_MB_, contact angle measured with milk broth; *θ*_W_, contact angle using water; *θ*_D_, contact angle using diiodomethane; *θ*_F_, contact angle using formamide; $\gamma_{s}$, total surface free energy; $\;y_{s}^{LW},$ apolar Lifshitz-van der Waals surface energy component; $\;\gamma_{s}^{AB}$, Lewis acid–base component; $\gamma_{s}^{+}$, electron-acceptor; $\gamma_{s}^{-}$, electron-donator. ^b^ Means ± standard errors of a minimum of 4 drops of each liquid on three individual pieces of material as well as ^c^ means ± standard deviation.

## Supplementary Materials

The following are available online at https://www.mdpi.com/2304-8158/10/3/611/s1, Figure S1: Schematic representation of the scraping technique used to detach biofilm cells from surface material coupons (PTFE, SS 320, SS 240). Scraping with a sterile pipette tip was performed four times vertically and horizontally across the entire coupon., Table S1: For cell detachment, scraping and sonication were compared as mean log CFU cm^−2^ of *M. lacticum* and *S. capitis* biofilms at 24 h, 48 h, 144 h, and 192 h on SS and PTFE surfaces ^a^.
