# Supplementary material for: Biofilm-Forming Ability of Microbacterium lacticum and Staphylococcus capitis Considering Physicochemical and Topographical Surface Properties
Source: Foods. 2021 Mar 13;10(3):611. doi: 10.3390/foods10030611 (PMC8001712; doi:10.3390/foods10030611)
Supplement: Supplementary file 1 [file foods-10-00611-s001.zip › foods-1125477-SI/SupplementaryTable1.pdf]

Table S1: For cell detachment, scraping and sonication were compared as mean log CFU cm^{-2} of *M. lacticum* and *S. capitis* biofilms at 24 h, 48 h, 144 h, and 192 h on SS and PTFE surfaces. [a]

	M. lacticum			*S. capitis*		
Growth time [h]	SS 320	SS 240	PTFE	SS 320	SS 240	PTFE
	Mean colony count [log CFU cm^{-2}] [b]					
	Cell detachment by scraping					
24	8.3 ± 0.2	8.2 ± 0.3	8.3 ± 0.3	6.8 ± 0.3	6.6 ± 0.2	6.2 ± 0.3
48	8.3 ± 0.2	8.2 ± 0.2	8.3 ± 0.2	7.0 ± 0.4	7.2 ± 0.4	6.0 ± 0.2
144	8.7 ± 0.1	8.8 ± 0.0	8.8 ± 0.2	7.9 ± 0.2	8.0 ± 0.1	7.8 ± 0.3
192	8.6 ± 0.1	8.7 ± 0.1	8.6 ± 0.1	7.8 ± 0.1	8.0 ± 0.2	7.9 ± 0.1
	Cell detachment by sonication					
24	8.0 ± 0.1	7.7 ± 0.0	8.0 ± 0.6	7.5 ± 0.3	7.4 ± 0.4	7.5 ± 0.5
48	7.8 ± 0.2	8.0 ± 0.3	8.0 ± 0.1	7.4 ± 0.3	7.5 ± 0.2	7.8 ± 0.1
144	8.6 ± 0.1	8.5 ± 0.1	8.5 ± 0.1	8.4 ± 0.1	8.3 ± 0.0	8.0 ± 0.0
192	8.5 ± 0.1	8.5 ± 0.1	8.4 ± 0.1	8.4 ± 0.1	8.2 ± 0.1	7.7 ± 0.1

[a] SS 320 (= Stainless steel with a 320 grit), SS 240 (=stainless steel with a 240 grit), PTFE (= polytetrafluoroethylene, Teflon ®)

[b] Means ± standard deviation of three independent experiments.
