# Supplementary figures and images for: Biofilm-Forming Ability of Microbacterium lacticum and Staphylococcus capitis Considering Physicochemical and Topographical Surface Properties
Source: Foods. 2021 Mar 13;10(3):611. doi: 10.3390/foods10030611 (PMC8001712; doi:10.3390/foods10030611)

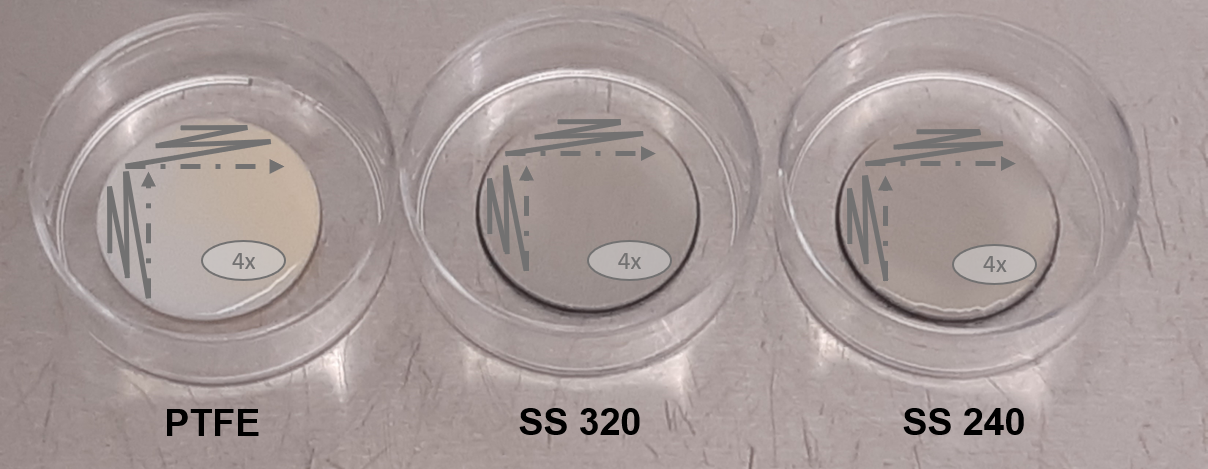

Supplement: Supplementary file 1 [file foods-10-00611-s001.zip › foods-1125477-SI/Supplemental_SupplementaryFigure1.tif]
